# Levels of pretreatment blood lipids are prognostic factors in advanced NSCLC patients treated with anlotinib

**DOI:** 10.1186/s12944-021-01596-5

**Published:** 2021-11-20

**Authors:** Mengqiu Tang, Chao Song, Yaowen Zhang, Xiaoyu Xu, Chen Wang, Zhanchun Zhang, Tian Chen

**Affiliations:** 1 Department of Radiation Oncology, Ningbo Medical Center Lihuili Hospital, Ningbo University, Ningbo, China; 2 Department of Surgery, Yuyao Maternity and Child Health Care Hospital, Ningbo, China; 3 Department of Cardiovascular Medicine, Ningbo Medical Center Lihuili Hospital, Ningbo University, Ningbo, China; 4 Department of Gastroenterology, Ningbo Medical Center Lihuili Hospital, Ningbo University, Ningbo, China

**Keywords:** Triglycerides (TG), Total cholesterol (TC), Low density lipoprotein (LDL), High density lipoprotein (HDL), Anlotinib, Non-small cell lung cancer (NSCLC), Prognostic factor, Prediction model

## Abstract

**Background:**

Anlotinib, a small molecule for multi-target tyrosine kinase inhibition, is the third or further line of defense for treatment of non-small cell lung cancer (NSCLC). Findings from an ALTER0303 phase III trial revealed that this drug confers significant survival benefits in patients. Although numerous inflammatory biomarkers have been shown to play vital roles in treatment, the clinical significance of blood lipid levels before treatment has not been evaluated. Here, this research aims to explore the relationship between blood lipids and efficacy of anlotinib, with a view of generating insights to guide future development of convenient and individualized treatment therapies.

**Methods:**

This study analyzed basal blood lipids levels, including triglycerides (TG), total cholesterol (TC), low density lipoprotein (LDL), and high density lipoprotein (HDL), among other variables before treatment, in 137 patients with advanced NSCLC who received anlotinib as third or further-line treatment at the Ningbo Medical Center Lihuili Hospital, between July 2018 and December 2020. We determined the best cut off value for predicting treatment responses, generated survival curves using the Kaplan–Meier method, then applied univariate and multivariate Cox regression analyses to assess predictors of survival.

**Results:**

The entire study population recorded median progression-free survival (PFS) and overall survival (OS) of 4 (95% CI 3.142–4.858) and 8.3 (95% CI 6.843–9.757) months, respectively. Researchers observed statistically significant differences across subgroups, between blood lipid indexes with different efficacies, except in the HDL subgroup. The low disease control rate (DCR) was associated with significantly elevated TG, TC and LDL levels (*P* = 0.000). Multivariate analysis demonstrated that elevated TC and LDL levels were independently associated with poor PFS or OS (*P* ≤ 0.003). Then, we established a prediction model, and set high TC or high LDL as the risk factor, respectively. There were significant differences in PFS (*p* = 0.000) and OS (*p* = 0.012) between 0 and ≥ 1 scores.

**Conclusions:**

Prior to anlotinib therapy, TC and LDL levels, are independent prognostic indicators for patients with advanced NSCLC treated with this drug as a third or further-line treatment option. In addition, a risk score of 0 was attributed to a combination of low TC and low LDL, and these patients were exhibited excellent efficacies and survival rates.

## Introduction

For decades, the rate of non-small cell lung cancer (NSCLC) has ranked highest among malignant tumors, with an overall 5-year survival rate of 20% [1, 2]. Most clinical diagnoses are performed when NSCLC is in advanced stages, which makes its treatment a challenge. Generally, treatment of NSCLC is stage specific [3, 4]. When not contraindicated, patients with stage I or II NSCLC can be effectively treated by surgical resection, with good prognostic outcomes. However, those with stage III or advanced NSCLC are subjected to local or systemic treatments, including targeted therapy, radiotherapy, chemotherapy or immunotherapy. These treatment options have shown excellent efficacies in patients who missed the opportunity for operation surgical resection [5–8]. Targeted therapies that are based on important driving genes for mutations have achieved excellent survival outcomes in patients with advanced NSCLC [9–12]. However, a large number of patients without gene mutations and those who are refractory to the targeted therapies exhibit poor clinical outcomes, necessitating the development of optimum treatment options to supplement the existing third-line options. Inconsistencies in third line treatment methods, as recommended by the NCCN, have shown limited therapeutic efficacies for NSCLC [13]. In China, an antiangiogenic therapy (anlotinib) is preferred as a third-line therapeutic option for advanced NSCLC [14]. Results from phase II and III clinical trials have shown that the novel vascular-targeting agent (anlotinib) prolongs progression-free survival (PFS) for NSCLC patients by another 4 months relative to the placebo. Besides, the findings indicated that the drug confers an overall disease control rate (DCR) of 81%, while the overall response rate (ORR) is only 9.2%. Moreover, the shortest and longest response durations for patients who achieved DCR were 1.5 and at least 18 months, respectively [14–17]. Considering the huge differences in efficacies of anlotinib, this study sought to identify biomarkers that regulate this phenomenon in order to improve prediction for the efficacy of anlotinib.

In advanced NSCLC, anlotinib is a small molecule multi-target tyrosine kinase inhibitor of vascular endothelial growth factor receptor 1–3 (VEGFR1–3), fibroblast growth factor receptor 1–4 (FGFR1–4), and platelet-derived growth factor receptor α-β (PDGFRα-β), among others [18, 19]. It plays a crucial role as a third or further line of targeted therapy and has shown excellent efficacy in treating NSCLC during clinical trials. Anlotinib has also been associated with various adverse reactions, including hyperlipidemia among other symptoms [15, 17]. These findings imply that, due to their roles in angiogenesis, blood lipid levels are potential predictive biomarkers for anlotinib therapy. Some previous studies have demonstrated that some elements, such as cluster of differentiation 31-labelled (CD31-labelled) activated circulating endothelial cells, levels of kallikrein-related peptidase 5 (KLK5) and L1 cell adhesion molecules (L1CAM), post-treatment hyperlipidemia, post-treatment hypertension status as well as pre-treatment Eastern Cooperative Oncology Group (ECOG) scores, are potential biomarkers for predicting anlotinib therapy in NSCLC patients [19–22]. However, clinical assessments of these genetic and cytological factors are expensive and inconvenient. Moreover, potential clinical associations between blood lipid levels before treatment and efficacies of anlotinib in NSCLC have not been evaluated. Therefore, this study analyzed the differences in basal lipid levels during patient survival as well as the effective ratio, and found that these parameters are significant predictors that can be used to guide future individualized treatment.

## Methods

### Patient recruitment and selection criteria

The present study retrospectively reviewed data from advanced NSCLC patients, who received anlotinib as a third or further-line treatment at the Ningbo Medical Center Lihuili Hospital, between July 2018 and December 2020. Participants were included if they: i. Were pathologically diagnosed with stage IV NSCLC (recurrent or metastatic); ii. Had an ECOG score of 3 or below; iii. Had no history of heart disease, renal or liver failure, or other contraindications to targeted therapy; iv. Underwent treatment with anlotinib as monotherapy more than 2 weeks after at least two previous lines of therapy for advanced disease; and v. Their treatment outcomes were assessed according to the Response Evaluation Criteria in Solid Tumors (RECIST) 1.1 criteria. Conversely, participants were excluded if they; i. Exhibited no form of hypolipidemic therapy prior to treatment; and ii. Had a baseline body mass index (BMI) score higher than 30 at baseline. Finally, a total of 137 patients conformed to the aforementioned criteria and were enrolled in the study.

### Participant information

Prior to anlotinib, this current study collected each patient’s basic information, including age, gender, and tumor stage among others, and also included patients whose baseline laboratory lipid information, namely TC, TG, LDL, and HDL, were available within 1 month prior to receiving anlotinib. The unit of lipids was mmol/L.

### Statistical analysis

Categorical variables were compared using either a chi-square or Fisher’s exact test. The best cut off value and AUC for the receiver operating characteristic curve (ROC) were determined for progression results. Overall survival (OS) time was defined as the time from anlotinib administration to death or final follow-up date, whereas PFS denoted the time from anlotinib administration to progressive disease (PD) or death resulting from any cause. Both OS and PFS were calculated using the Kaplan–Meier method, and the resulting survival curves compared using the log rank test. Hazard ratios (HR) were estimated using the Cox regression analysis method. ORR was the sum of partial response (PR) and complete response (CR), while DCR was equal to the sum of ORR and stable disease (SD). Correlations between optimal treatment efficiency (%) and baseline lipid stratification were determined using the Chi square test, with multivariate analysis for the most significant variables performed using the Cox regression model. Statistical analyses were counted by R version 3.3.3 (R Foundation for Statistical Computing, Vienna, Austria) and SPSS software (version 20.00, SPSS, Chicago, IL). Statistical significance was set at *P* < 0.05.

## Results

### Patients’ physical and clinical characteristics

A summary of physical and clinical characteristics for the 137 patients included in this research are listed in Table 1. Briefly, patients’ ECOG scores ranged from 0 to 3, and none of them had been administered with treatment to either increase or decrease their blood lipid levels. The median age was 62 years old. Clinic-pathological diagnosis revealed that all patients presented more than 4 tumor stages. Squamous and non-squamous cell carcinoma accounted for 51.8 and 48.2%, respectively, and the median line of treatment using anlotinib was third-line. The other three items alongside their median values are also listed in Table 1.

**Table 1:** Physical and clinicopathological characteristics of the 137 patients included in the present study

Patient characteristics	N = patients (%)
All	137
Age (years)	
< 65	83 (60.5%)
≥ 65	54 (39.5%)
Gender	
Male	99 (72.3%)
Female	38 (27.7%)
Histology	
Squamous	71 (51.8%)
Non-squamous	66 (48.2%)
Performance Status (ECOG)	
0–1	95 (69.3%)
2–3	42 (30.7%)
Driver gene	
Wild	108 (78.8%)
Mutation	29 (21.2%)
Metastasis sites	
≤ 3	73 53.3%)
> 3	64 (46.7%)
Line(s) of treatment	
3	78 (56.9%)
> 3	59 (43.1%)

### Optimal cut-off values for lipids

**Fig. 1:**
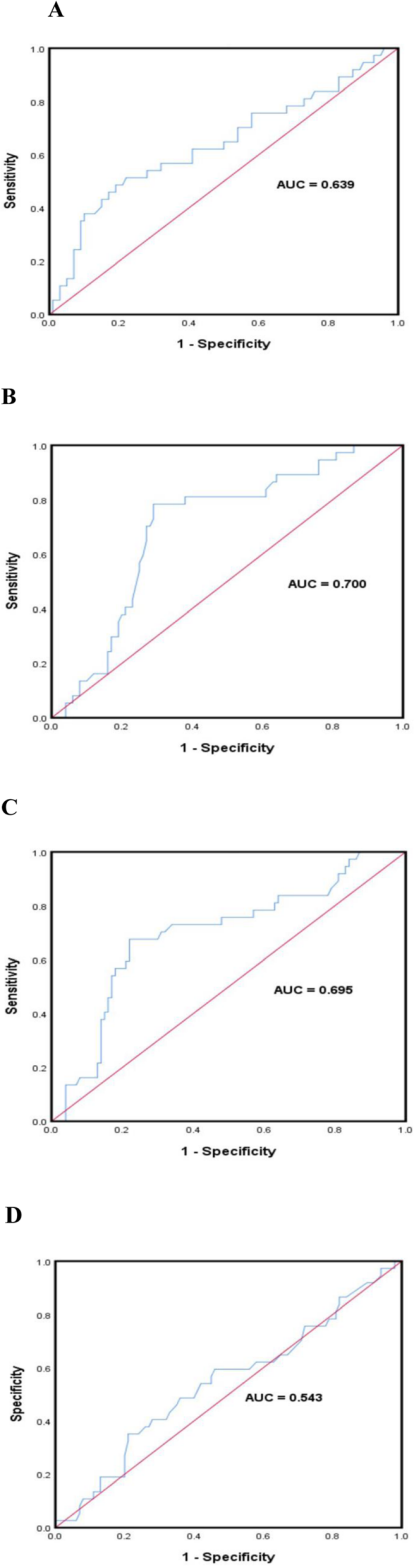
Receiver operating curves showing response to treatment and optimum cut-off values for triglyceride (**A**), total cholesterol (**B**), low density lipoprotein (**C**), and high density lipoprotein (**D**)

The ROC was generated to determine the optimal cut-off values for the aforementioned blood lipids in all patients. The optimal cut-off value for TG was 1.82 with an area under curve (AUC) of 0.639 (*P* = 0.013, 95% CI: 0.527–0.751), that for TC was 4.77 with an AUC of 0.700 (*P* = 0.000, 95% CI: 0.606–0.795), whereas those for LDL and HDL were 2.965 (*P* = 0.000, 95% CI: 0.592–0.797) and 1.095 (*P* = 0.441, 95% CI: 0.431–0.654), respectively, with corresponding AUC values of 0.695, 0.543. A summary of ROC curves is presented in Fig. 1.

### Curative effect analysis

No patient from the analyzed cohort achieved CR (0%), thus the ORR (4%) was thought to be equal to the PR value (4%). SD was the main response. Curative effects among different lipids groups layered were calculated based on their respective optimal cut-off values (Table 2). For TG, a total of 100 patients reached DCR, with the low group (TG < 1.82) comprising a significantly higher proportion (59.1%) relative to the high group (13.9%, *P* = 0.000). Conversely, the high group exhibited the highest proportion of PD (13.9%), which was significantly different from those obtained in PR and SD (*P* = 0.002). Findings from LDL corroborated those of TG and TC (Table 2). However, in the HDL group, there were no significant differences in curative effects.

**Table 2:** Associations among the four lipids with treatment response

Response	n	%	TG			TC			LDL			HDL		
			< 1.82	≥1.82	*P* value	< 4.77	≥4.77	*P* value	< 2.965	≥2.965	*P* value	< 1.095	≥1.095	*P* value
CR	0	0												
PR	6	4	5 (3.6%)	1 (0.7%)	0.002	4 (2.9%)	2 (1.4%)	0.000	5 (3.6%)	1 (0.7%)	0.000	2 (1.4%)	4 (2.9%)	0.218
SD	94	68.6	76 (55.5%)	18 (13.1%)		67 (48.9%)	27 (19.7%)		73 (53.3%)	21 (15.3%)		52 (38%)	42 (30.1%)	
PD	37	27	19 (13.9%)	18 (13.1%)		8 (5.8%)	29 (21.2%)		12 (8.8%)	25 (18.2%)		15 (10.9%)	22 (16.1%)	
ORR	6	4												
DCR	100	72.6	81 (59.1%)	19 (13.9%)	0.000	71 (51.8%)	29 (21.2%)	0.000	78 (56.9%)	22 (16.1%)	0.000	54 (39.4%)	46 (33.6%)	0.162

The unit of measurement for lipids is mmol/L

*CR* complete response, *PR* partial response, *SD* stable disease, *PD* progressive disease, *ORR* overall response rate, *DCR* disease control rate, *TG* triglyceride, *TC* total cholesterol, *LDL* low density lipoprotein, *HDL* high density lipoprotein

### Prognostic values of layered baseline lipid levels in the overall population

**Fig. 2:**
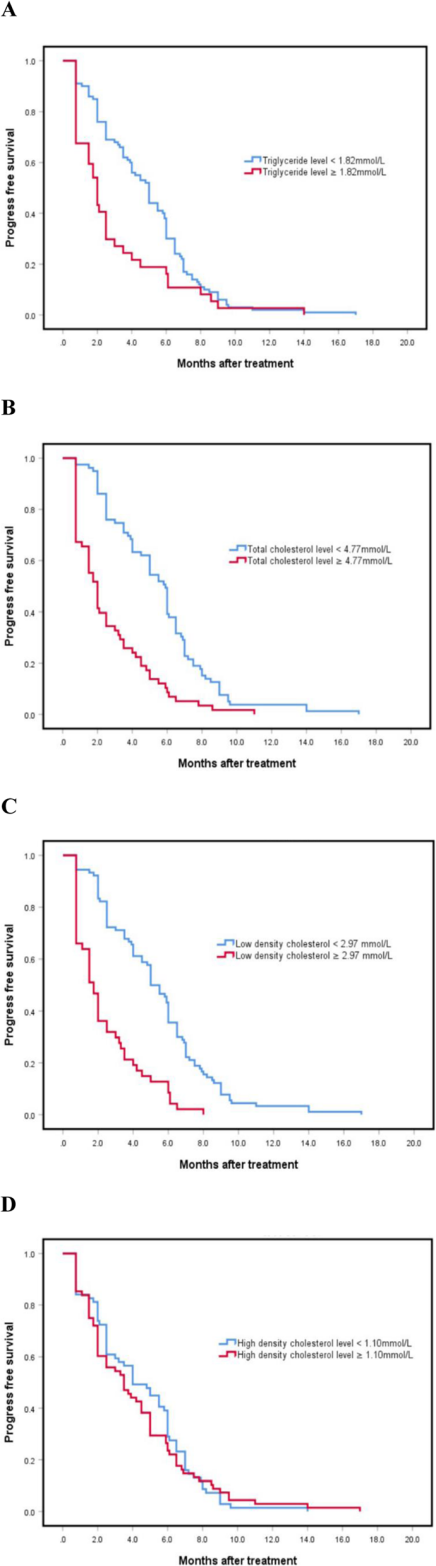
Relationship between triglyceride, total cholesterol, low density lipoprotein, and high density lipoprotein with progression-free survival rates (*P*_A_ = 0.004, *P*_B_ = 0.000, *P*_C_ = 0.000, *P*_D_ = 0.619)

**Fig. 3:**
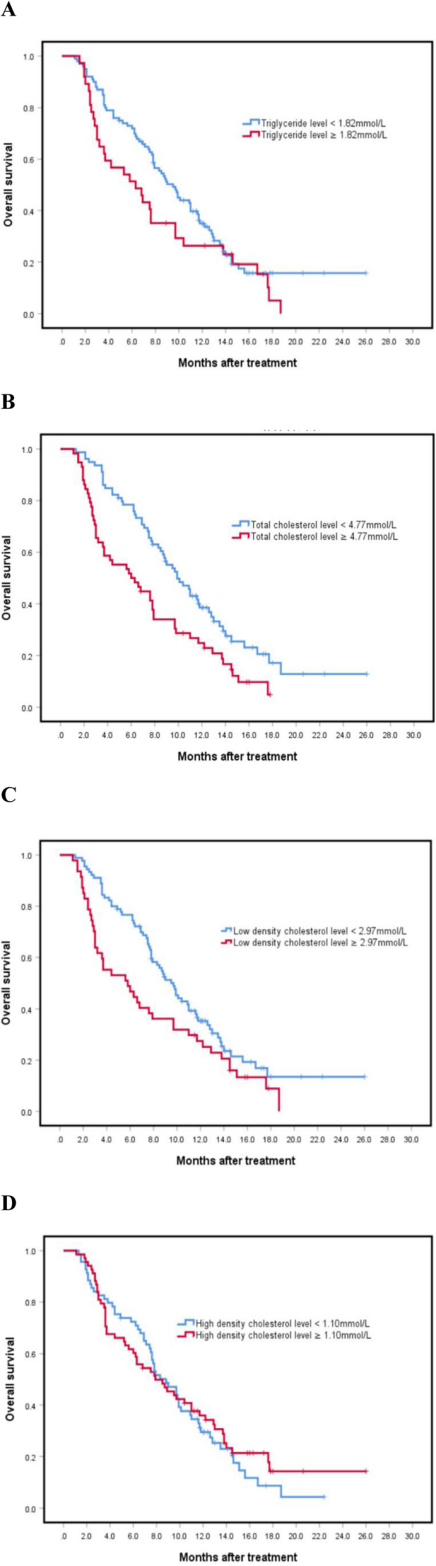
Relationship between triglyceride, total cholesterol, low density lipoprotein, and high density lipoprotein with overall survival rates (*P*_A_ = 0.084, *P*_B_ = 0.003, *P*_C_ = 0.029, *P*_D_ = 0.622)

The study population had a median follow-up time of 16.3 months, and all patients exhibited recurrence. The median PFS and OS for the study participants were 4 (95% CI 3.142–4.858) and 8.3 (95% CI 6.843–9.757) months, respectively. Univariate analyses showed that TG, TC and LDL were significant risk factors for PFS, while TC and LDL were risk factors for OS (Table 3). By integrating the significant risk factors into multivariate analysis, it was found that high TC and high LDL were independently associated with poor PFS (Table 4). With regards to TC (Figs. 2B and 3B), the high TC group exhibited shorter median PFS and OS rates, at 2 and 6 months, respectively, compared to those in the low group, namely 5.9 and 9.9 months, respectively (*P*_PFS_ = 0.000, *P*_OS_ = 0.003). Moreover, the high LDL group exhibited significantly shorter median PFS (Fig. 2**C**) and OS (Fig. 3**C**) compared to the low group (1.75 months vs 5 months, *P*_PFS_ = 0.000; 5.8 months vs 9.7 months, *P*_OS_ = 0.029). The high TG group exhibited significantly shorter median PFS (Fig. 2**A**) compared to the low group (2.0 months vs 5.0 months, *P* = 0.004) while the high and low TG groups had no significant differences in OS (Fig. 3**A**). Notably, there were no significant differences in PFS (Fig. 2**D**) or OS (Fig. 3**D**) between the high and low HDL groups (Table 4).

**Table 3:** Results of univariate analysis of factors associated with progression-free (PFS) and overall survival (OS) rates

Variable (*N* = 137)	PFS			OS		
	HR	95%CI	Univariate (*P* value)	HR	95%CI	Univariate (*P* value)
Age (years)	1.057	0.756–1.505	0.713	0.897	0.508–1.324	0.564
< 65						
≥ 65						
Gender	1.162	0.797–1.694	0.435	1.067	0.697–1.634	0.764
Male						
Female						
Histology	1.198	0.855–1.679	0.294	1.165	0.799–1.700	0.428
Squamous						
Non-squamous						
Performance Status (ECOG)	1.168	0.810–1.584	0.405	1.453	0.975–2.165	0.066
0–1						
2–3						
Driver gene	1.002	0.663–1.515	0.993	1.147	0.726–1.811	0.557
Wild						
Mutation						
Metastasis sites	0.932	0.667–1.316	0.707	0.997	0.685–1.453	0.989
≤ 3						
> 3						
Line(s) of treatment	1.063	0.758–1.494	0.725	0.768	0.523–1.127	0.178
3						
≥ 3						
TG	1.689	1.152–2.475	0.007	1.431	0.949–2.159	0.088
< 1.82						
≥ 1.82						
TC	2.647	1.655–3.777	0.000	1.773	1.213–2.592	0.003
< 4.77						
≥ 4.77						
LDL	3.056	2.091–4.495	0.000	1.532	1.040–2.257	0.031
< 2.965						
≥ 2.965						
HDL	1.085	0.773–1.522	0.637	0.910	0.623–1.328	0.624
< 1.095						
≥ 1.095						

The unit of measurement for lipids is mmol/L

*HR* hazard ratio, *CI* confidence interval, *TG* triglyceride, *TC* total cholesterol, *LDL* low density lipoprotein, *HDL* high density lipoprotein

**Table 4:** Results of multivariate analysis of significant factors associated with progression-free survival (PFS)

Variable (N = 137)	PFS		
	HR	95%CI	Multivariate (*P* value)
TC	1.841	1.187–2.857	0.006
< 4.77			
≥ 4.77			
LDL	2.133	1.336–3.406	0.002
< 2.965			
≥ 2.965			

The unit of measurement for lipids is mmol/L

*HR* hazard ratio, *CI* confidence interval, *TC* total cholesterol, *LDL* low density lipoprotein

### The prognostic prediction model by TC and LDL value

**Fig. 4:**
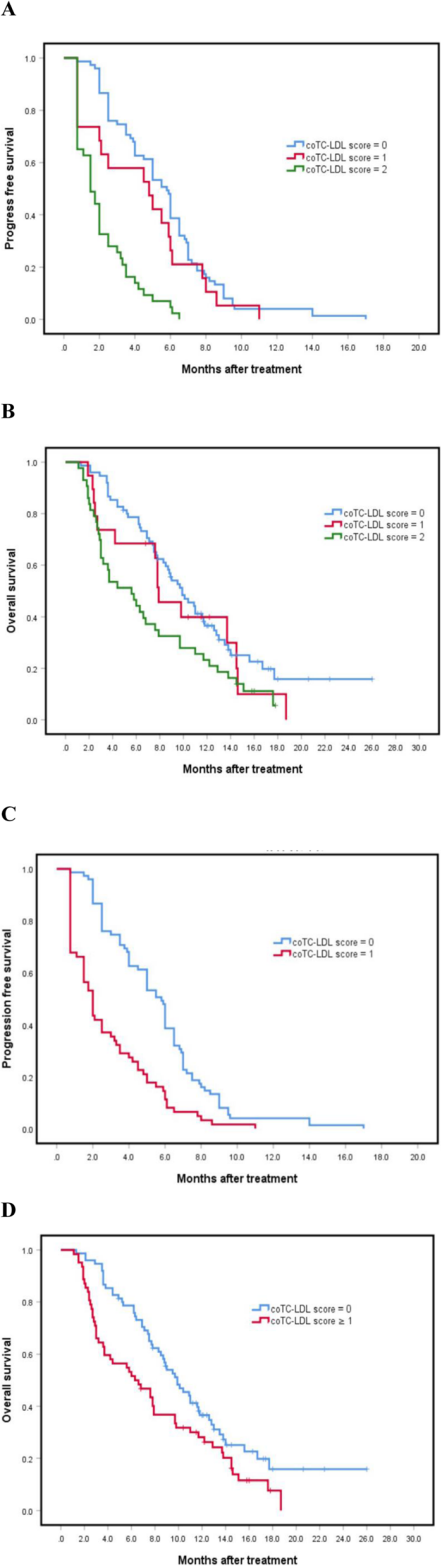
Relationship among the combination of total cholesterol and low density lipoprotein (coTC-LDL) scoring groups with PFS and OS (*P*_A_ = 0.000, *P*_B_ = 0.017, *P*_C_ = 0.000, *P*_D_ = 0.012)

The results incorporated the significant factors (baseline TC and LDL values) into multivariate analysis to identify the independent prognostic factors. The high value was equivalent to a risk factor, and in the presence of each risk factor, the patients’ risk score was raised by 1. Based on this, patient’s scores ranged from 0 (extremely favorable) to 2 score (extremely unfavorable). Scores of 0, 1 and 2 were significantly associated with PFS (*P* = 0.000, 95%CI 3.142–4.858) and OS (*P* = 0.017, 95%CI 6.843–9.757). Specifically, 0 score was associated with superior survival rates, relative to 1 and 2 scores (Table 5). However, survival rates showed a cross connection among the three groups (Fig. 4A, B). Additionally, in grouped comparisons, the 0 score did not exhibit significant differences when compared to score 1 in terms of mPFS(*P* = 0.198). Therefore, this paper combined scores 1 and 2 groups into a high score group (≥ 1 score), then re-calculated survival outcomes between 0 score and ≥ 1 score groups. These two groups had significant differences in PFS and OS (Fig. 4C and D).

**Table 5:** Median progression-free survival (mPFS) and median overall survival (mOS) for different scores in patients stratified according to presence of different independent prognostic factors obtained from multivariate analysis (TC and LDL)

Prognosis	Score			95%CI	*P* value
	0(*n* = 75)	1(*n* = 19)	2(*n* = 43)		
mPFS (months)	5.8	4.8	1.5	3.142–4.858	0.000
mOS (months)	9.9	7.9	5.6	6.843–9.757	0.017

The unit of measurement for lipids is mmol/L

*TC* total cholesterol, *LDL* low density lipoprotein, *CI* confidence interval

A score of 0 represents low TC (< 4.77) and low LDL (< 2.965) at baseline; a score of 1 denotes low TC (< 4.77) and high LDL (≥ 2.965) or low LDL (< 2.965) and high TC (≥ 4.77) at baseline; whereas 2 represents high TC (≥ 4.77) and high LDL (≥ 2.965) at baseline

## Discussion

Angiogenesis is vital in tumorigenesis and tumor development, therefore, inhibition of angiogenesis is vital for tumor control [23, 24]. Particularly, vascular endothelial growth factors (VEGFs) and VEGFRs are key family members of angiogenesis regulators, whose combinations have been shown to promote angiogenesis [25–27]. Anlotinib, an oral multi-targeted tyrosine kinase receptor inhibitor, has been shown to actively regulate anti-angiogenesis and selectively inhibit VEGFR (2/3), FGFR (1–4), and PDGFR (α/β), as well as other targets [17–19]. Numerous studies have demonstrated the role of VEGFs in lipid and lipoprotein metabolism. Particularly, VEGF-A has been shown to be significantly upregulated in heart and blood vessel diseases [28, 29], whereas VEGF-B is basically homologous with VEGF-A [30]. In the past, VEGF-C/D were thought to be associated with lymphangiogenesis, with several studies reporting that VEGF-D plays a crucial role in lipid metabolism via its endothelial cell receptors VEGFR-2/3 [31, 32]. Furthermore, VEGF-A, VEGF-D, VEGFR-1 and VEGFR-2 were found to be elevated in hyperlipidemic rabbits [33]. Despite the fact that VEGFA-D and VEGFR1–4 belong to the same receptor family, their functions in blood lipids are complicated and contradictory while their regulation is controlled by unknown factors. Cancer cells are known to exhibit elevated lipid and cholesterol levels, which are satisfied by increasing food intakes or external carbohydrates, lipoelasticity or lipid synthesis [33–35]. This aberrant lipid metabolism not only influences primary tumors, but also affects exogenous lipid production by the tumor microenvironment, thereby predisposing body tissues to malignancy [36, 37]. Based on these findings, it is evident that lipid metabolism processes are often upregulated in cancer.

In this study, high baseline lipid levels, mainly TC and LDL levels, were associated with short survival times for patients and had an effect on the efficacy of anlotinib therapy. Specifically, this study participants exhibited median OS and PFS times of 8.3 and 3.1 months, respectively, which were slightly shorter than the corresponding 5 (PFS) and 9.6 (OS) months from ALTER0303 [15]. This may be attributed to the fact that the patients included in this study were all diagnosed with stage 4 cancer and were treated with anlotinib as a third or further line, relative to those in ALTER0303 who were at stage 3 or using anlotinib as a second-line therapy. Several factors, such as ECOG scores before treatment as well as hyperlipidemia and hypertension status after treatment have been shown to play some predictive roles in the prognosis of anlotinib therapy, mainly due to occurrence of adverse reactions [19, 20]. However, previous studies had not investigated the association between combined basal lipid levels and prognosis. In this research, there were no significant differences in survival outcomes among different ECOG scores. This could be because, in order to eliminate data skew, this paper divided ECOG scores 0–1 into a group and 2–3 into another group, which had resulted from the lack of score 0 patients. However, for previous studies, because of the absence of score 3, they assigned ECOG score 0 into a group and 1–2 into another group. In this study, we initially analyzed the association between anlotinib efficacy and different basal lipids levels. Results from multivariate analysis showed that elevated TC and LDL levels are independent predictors for poor PFS. Moreover, survival data confirmed that high basal TC and LDL levels might result in poor anlotinib efficacies in NSCLS patients. This retrospective study enrolled patients with comparatively advanced disease status, which may have led to very poor natural prognosis. In addition, different posterior line treatments may have influenced the overall survival rates. Based on the reason for the divergence of multivariate analysis registering as three factors in PFS but just one factor in OS, a scoring system with scores ranging from 0 to 2 involving basal TC and LDL levels was established. In this model, we expected that a high value would represent a high risk and anticipated the significant differences. Although there were significant differences in PFS (*P* = 0.000) and OS (*P* = 0.017) among the 3 groups, in the PFS curve, scores of 0 and 1 were so close together that they generated a negative result between them (*P* = 0.198) during stratified analysis. Given that the small sample size might have led to the lack of score 1 patients, the present study reconsidered a high score group (≥ 1 score) comprising both 1 and 2 score groups, and found statistically significant differences in PFS (*P* = 0.000) between 0 and ≥ 1 scores, as well as in OS (*P* = 0.012).

### Strength and limitations

This retrospective study proved that the baseline blood lipid levels were directly correlated with treatment responses and prognosis in advanced NSCLC patients treated with anlotinib. Based on calculated results, this paper hypothesized that the basal TC and LDL levels are potential predictors for the efficacy of anlotinib in NSCLC patients. However, this study has some limitations. First, all patients were in advanced NSCLC stages, suggesting that many factors may have affected the observed outcomes. In addition, their diets and nutritional status might have influenced the recorded lipid levels. Second, it is possible that subsequent therapy after anlotinib, which involved radiochemotherapy and immunotherapy, may have potentially affected prognosis. Last, the sample size was relatively small, which may have resulted in minimal PR and 1 score group, thereby introducing a potential bias. Moreover, studies should aim at validating the established model using independent data sets.

## Conclusion

In summary, basal TC and LDL levels are potential biomarkers for evaluating patient responses to anlotinib therapy during treatment of advanced NSCLC. Patients with elevated baseline TC and LDL levels, prior to anlotinib therapy, exhibited inferior curative efficacies and survival rates than those in the low group. Taken together, these findings indicate that changes in blood lipid levels during anlotinib treatment are potential prognostic factors, and can be used to inform personalized treatment for advanced NSCLC.

## Data Availability

All data used and analyzed during this study will be available from the corresponding author on reasonable request.
